# Iron-Induced Fibrin in Cardiovascular Disease

**DOI:** 10.2174/15672026113109990016

**Published:** 2013-08

**Authors:** Boguslaw Lipinski, Etheresia Pretorius

**Affiliations:** 1 Joslin Diabetes Center, Harvard Medical School, Boston, MA 02215, USA; 2 Department of Physiology, Faculty of Health Sciences, University of Pretoria, Arcadia 0007, South Africa

**Keywords:** Cardiovascular disease, fibrinogen, free radicals, inflammation, iron, parafibrin, thrombosis.

## Abstract

Accumulating evidence within the last two decades indicates the association between cardiovascular disease (CVD) and chronic inflammatory state. Under normal conditions fibrin clots are gradually degraded by the fibrinolytic enzyme system, so no permanent insoluble deposits remain in the circulation. However, fibrinolytic therapy in coronary and cerebral thrombosis is ineffective unless it is installed within 3-5 hours of the onset. We have shown that trivalent iron (FeIII) initiates a hydroxyl radical-catalyzed conversion of fibrinogen into a fibrin-like polymer (parafibrin) that is remarkably resistant to the proteolytic dissolution and thus promotes its intravascular deposition. Here we suggest that the persistent presence of proteolysis-resistant fibrin clots causes chronic inflammation. We study the effects of certain amphiphilic substances on the iron- and thrombin-induced fibrinogen polymerization visualized using scanning electron microscopy. We argue that the culprit is an excessive accumulation of free iron in blood, known to be associated with CVD. The only way to prevent iron overload is by supplementation with iron chelating agents. However, administration of free radical scavengers as effective protection against persistent presence of fibrin-like deposits should also be investigated to contribute to the prevention of cardiovascular and other degenerative diseases.

## INTRODUCTION

Numerous epidemiological and large prospective studies have shown that hypercoagulability [1] and the increased blood level of fibrinogen (FBG) are important risk factors for cardiovascular disease [2-4]. It is generally agreed that the higher content of fibrinogen in plasma the greater the chance of thrombus formation, therefore its level should be maintained as low as possible. It should be remembered, however, that critical factor in thrombosis is not the absolute amount of FBG, but the fate of its thrombin-generated product, fibrin. Under physiological conditions fibrin clots are gradually, albeit completely, removed from the site of vessel wall injury by the powerful fibrinolytic system of blood [5].

However, if for some reason the fibrinolytic system is inefficient, persisting thrombi will obstruct the flow of blood with all its pathological consequences. The best example of such a situation is the use of a thrombin-like enzyme of viper venom (Ancrod) for the prevention of thrombosis [6]. Thus, despite a complete conversion of all circulating FBG to fibrin, no persistent thrombi are produced due to the solid phase activation of fibrinolysis by fibrin [7]. Disseminated intravascular thrombosis occur only when fibrinolysis is inhibited by eg. *Aprotinin* [8].

The presence of fibrin-like material in atherosclerotic plaques was first observed over 150 years ago by Karl Rokitansky [8] and later confirmed by other researchers [1, 9-11]. In 1995 Elspeth Smith documented the existence of fibrinogen-related antigens (FRA) the insoluble fraction obtained from atherosclerotic intima [12]. According to this author FRA is present at the surface as well as deep inside the plaques thus providing scaffolding for migration and proliferation of smooth muscle cells. It should be emphasized that this type of insoluble FGB deposits are morphologically different from fibrin present in thrombi formed as a result of plaque raptures [13]. This fact obscures identification of fibrin-like material present inside the arterial wall, and may perhaps explain why the attention of researches were drawn away and directed to a more popular concept of the role of cholesterol [14].

The presence of insoluble FBG deposits in atherosclerotic plaques was also attempted to be explained by the loss of negative charge of the arterial wall of subjects with CVD. It was demonstrated that the amount of acid mucopolysaccharides (AMPS) extractable from the arterial intima was significantly lower in CVD patients as compared to young and healthy subjects [15]. According to this idea AMPS would form soluble complexes with blood fibrin monomers thus preventing their anchoring and deposition on the endothelial cells. However, this mechanism would require a chronic activation of intravascular blood coagulation that has still to be proven.

## THE ROLE OF IRON

Although atherosclerosis is also known to be associated with the inhibition of fibrinolysis, no specific mechanism and/or agent(s) have been identified. Studies by Undas and collaborators shed some light on this problem by showing that the susceptibility of fibrin clot to lysis is affected by the structure and permeability of fibrin network [16]. This phenomenon is compatible with the observed thrombolytic resistance in patients with coronary and/or cerebral thrombosis [17].

Another important fact is that there is a relationship between body iron overload and pathogenesis of numerous degenerative diseases, including atherosclerosis [18-23]. Particularly relevant is the extensive review by D.B.Kell on the role of free blood iron in various pathological conditions. [24]. In addition, links have been found to exist between iron body stores, cardiovascular risk factors and hypercoagulability [25, 26]. Moreover, in experimental models the infusion of trivalent iron salts was shown to cause diffused thrombosis [27]. It is a common belief that free blood iron, via the Fenton-like reaction, is responsible for so-called oxidative stress that, in turn, leads to atherosclerosis and related cardiovascular diseases [28]. Yet, despite this attractive, albeit simplistic, concept no effectiveness of antioxidant therapy has been demonstrated [29]. As a result numerous natural products (specifically polyphenols) are not being clinically tried because they had been labeled as antioxidants. This highly controversial and, in fact, damaging notion was dealt with in a recent article, which emphasized the importance of polyphenolic substances as iron chelating and free radical scavenging agents that may be neither oxidants nor antioxidants [30].

### Iron-Induced Conversion of Fibrinogen to Parafibrin

We have recently documented that trivalent iron ion (FeIII) generates in aqueous solutions powerful hydroxyl radicals that subsequently modify fibrinogen molecules converting them to insoluble fibrin-like polymer [31]. It should be emphasized that such a polymer is not only resistant to fibrinolytic dissolution, but also to proteolytic digestion, i.e. with chymotrypsin, that normally degrades fibrin(ogen) into smaller polypeptide fragments. Protein chemistry teaches us that undesirable molecular interactions in blood proteins are prevented by holding their hydrophobic groups inside the interior of protein tridimensional structures stabilized by *intra*-molecular disulfide bonds. Once these bonds are broken the polypeptide chains become unfolded with the exposure of hydrophobic domains which form *inter*-molecular bonds resulting in the formation of large aggregates. It is of great importance to note that such aggregates cannot be degraded by the proteolytic enzymes as is the case with human prion proteins [32] and bacterial hydrophobins [33]. Consequently, it is often very difficult to identify insoluble fibrin deposits in pathologically affected organs in various chronic diseases, because no antigen can be released into the liquid phase of the extracted tissues. In concordance with this, the presence of insoluble fibrin(ogen) deposits can only be demonstrated by a direct immunochemical staining of the tissue sections.

### Parafibrin as an Inflammation Inducer

**Fig. (1):**
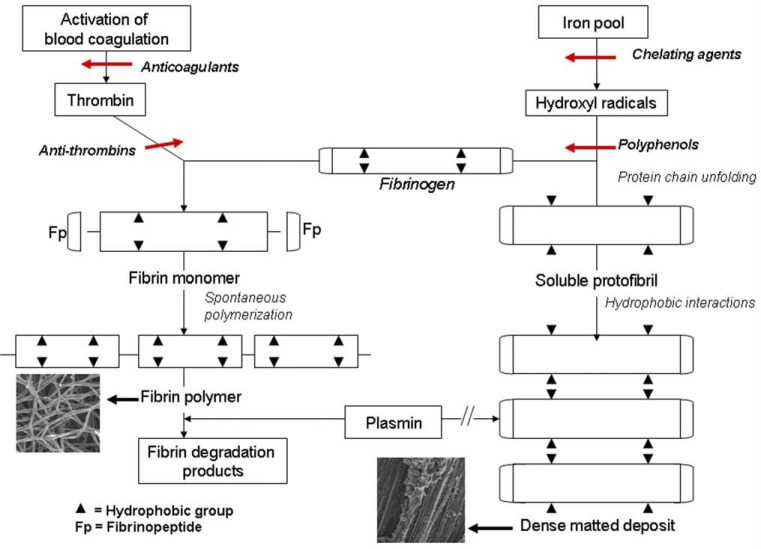
Conversion of plasma fibrinogen into insoluble polymers catalyzed by thrombin (left panel), and the formation of dense fibrin deposits generated with iron (right panel). By contrast to the enzymatically formed fibrin susceptible to fibrinolysis, the iron-induced fibrin polymer is remarkably resistant to proteolytic degradation. Reprinted with permission from Pol Arch Med Wewn. Vol.122, p.120 (Fig.6), 2012. ^31^

The resistance of fibrin clots to enzymatic degradation can now be explained by our finding of the alternative iron-induced mechanism of blood coagulation (Fig. **1**). According to this concept free iron of blood (Fe III) generates hydroxyl radicals, which in turn convert circulating FBG into an insoluble fibrin-like material ( or parafibrin) without the action of thrombin [31]. It should be strongly emphasized that this pseudo or parafibrin is one of very few proteins, such as prions [32] and bacterial hydrophobins [33], that are totally resistant to enzymatic proteolysis. As a consequence such a dense fibrin polymer acquires the features of a foreign body and attracts macrophages resulting in a permanent state of inflammation known to be associated with atherosclerosis [34-37]. Also it is of interest to note the reports on the relationship between inflammation and blood coagulation. Moreover there are numerous experimental and clinical studies that indicate the relationship between inflammation, iron overload and cardiovascular diseases [20, 38-40].

### Protective Mechanisms Against Iron-Induced Pathology

As shown in Fig. (**1**) the intravascular formation of modified fibrin can be inhibited at two stages. First, and perhaps the most important, is the inactivation of free iron, usually achievable by the administration of a variety of iron chelators [24, 41-47], as well as by other means of the reduction of body iron stores [48]. If this fails, the next step is elimination of hydroxyl radicals by means of a number of natural and/or synthetic scavengers. The hydroxyl radical scavenging reaction occurs by virtue of aromatic hydroxylation, as exemplified by the reaction with salicylic acid known to prevent inflammation and its consequences [47]. This reaction is believed to be responsible for the beneficial health effects of polyphenolic substances present in fruits and vegetables of the so-called Mediterranean diet [49]. Small molecular weight phenolic compounds such as chlorogenic acid, ferulic and coumaric acids, consumed with certain food products, become even more effective hydroxyl radical scavengers due to their enhanced absorption from the alimentary track. The larger molecules of polyphenols have to be first metabolized by the intestinal flora (probiotics) in order to achieve their *in vivo* health beneficial effect [50].

**Fig. (2):**
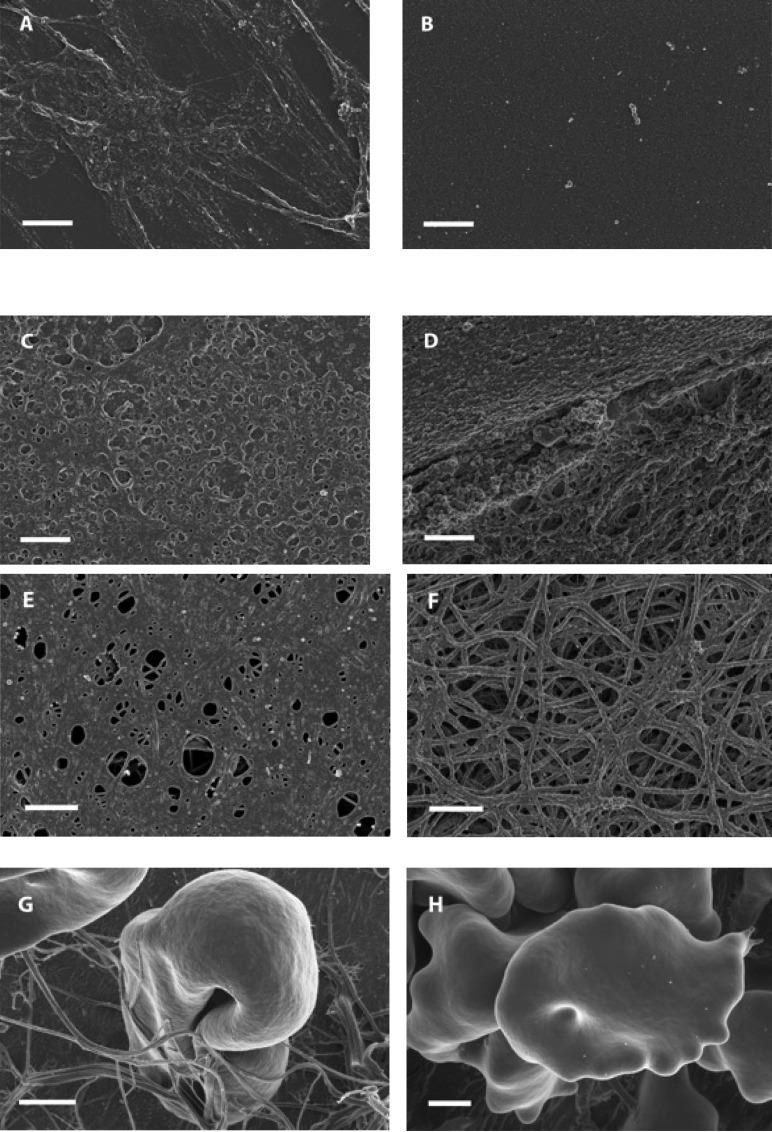
Effects of certain amphiphilic substances on the iron- and thrombin-induced fibrinogen (FGB) polymerization visualized USING scanning electron microscopy, **A.** Control: Purified fibrinogen (PF) and ferric chloride (FC); **B.** PF + Tween 20 + FC ; **C.** PF + high-density lipoprotein + FC; **D.** PF + low-density lipoprotein + FC; **E.** Low-albumin plasma (LAP)+ thrombin; **F.** LAP + purified human albumin + thrombin; **G.** Whole blood of a stroke patient; **H.** Normal whole blood + FC. Scale = 1 µm.

In concert with this observation it is the fact that the altered fibrin structure argued to be associated with cardiovascular disease (CVD) can be normalized by the pretreatment with hydrophilic substances e.g. high-density lipoprotein (HDL) [51] and/or human serum albumin known to be decreased in CVD [52-55]. The protective effect of such substances is documented here using SEM method for HDL (Fig. **2C**), and for a non-ionic detergent Tween 20 (Fig. **2A** and **B**). These results stand in contrast with the potentiation of iron-induced dense parafibrin formation exerted by low density lipoprotein (Fig. **2D**). It is also possible that the health beneficial effect of human serum albumin (HSA), a highly hydrophilic protein, is due to the restoration of normal fibrin strands generated with thrombin (Fig. **2E** and **F**). Therefore, it can be concluded that it is not just iron homeostasis, but the blood content of hydrophilic and polyphenolic agents that is important in the prevention of atherosclerosis.

## THE ROLE OF RED BLOOD CELLS

Another pathologic process leading to atherosclerosis is the impaired blood flow caused by the intravascular aggregation of red blood cells (RBC). Although its mechanism is not completely understood, it is well known that the elevated erythrocyte sedimentation rate (ESR) is associated with inflammation and CVD. (56-59) We have shown for the first time that the abnormal RBC morphology induced by iron ions added to normal blood is strikingly similar to that observed in blood of stroke patients [60] as well as of subjects with diabetes mellitus [61]. These changes are shown here in Fig. (**2G**) (Stroke) and H (healthy blood with added ferric iron). It is argued that the close association between RBC and the modified fibrinogen molecules can be caused by the interaction between hydrophobic epitopes on the cell membranes and those of the soluble fibrin *protofibrils* generated with iron (Fig. **1**). This mechanism may explain significant reduction of blood flow [62] and increased blood viscosity in patients with thrombotic arterial disease [63]. The relationship between inflammation and elevated ESR was emphasized years ago by Zacharski and Kyle [64]. Finally, it should be noted that RBC aggregation and sedimentation were originally thought to be caused by blood soluble fibrin monomers by virtue of their interaction with hydrophobic epitopes on RBC membranes [65]. However, this concept was abandoned in view of the absence of any evidence of the link between chronic activation of intravascular blood coagulation and ESR. The concept of iron-induced parafibrin formation offers more plausible mechanism of the relationship between hemorheologic disturbances and inflammation.

## CONCLUSION

In conclusion, we postulate in this paper that the excess of blood free iron is responsible for the non-enzymatic generation of insoluble fibrin-like material (parafibrin) that, when deposited on the arterial wall, initiates inflammatory reactions. This pathological process, very different from the classical activation of blood coagulation, can be prevented by substances that chelate iron, scavenge hydroxyl radicals, and inhibit hydrophobic interactions in proteins. However, in view of the fact that, so far, there is no known agent or a biological process that can degrade parafibrin, an interdisciplinary research approach is needed to find an effective method for the elimination from the human body this unique inducer of chronic inflammation.
